# The ostomy leak impact tool: development and validation of a new patient-reported tool to measure the burden of leakage in ostomy device users

**DOI:** 10.1186/s12955-018-1054-0

**Published:** 2018-12-14

**Authors:** Beenish Nafees, Zenia M. Størling, Charlotte Hindsberger, Andrew Lloyd

**Affiliations:** 1 Nafees Consulting Limited, London, UK; 2 Coloplast A/S, Holtedam 3, 3050 Humlebæk, Denmark; 3 Acaster Lloyd Consulting Ltd, London, UK

**Keywords:** Leakage, Stoma, Quality of life, Measure, Ostomy

## Abstract

**Background:**

Leakage is a major concern for people who use a stoma, but people’s experience and its impact is not well understood. This study aimed to establish a definition of leakage through clinical and user input. This information was used to develop and validate a new measurement tool to understand the impact of leakage for people using a stoma appliance, in the UK, US, France, and Denmark.

**Methods:**

Participants were recruited from a panel of users, hosted by Coloplast, that includes people who currently use Coloplast products. Six clinicians and 41 users took part in concept elicitation interviews. The qualitative findings were used to draft items. A panel of clinical experts was organized to develop and validate items (*N* = 6). Cognitive debrief interviews were conducted with five users in each country, which resulted in removing some items and revising the measure. A psychometric validation was conducted with 340 people in four countries whereby participants were asked to complete a series of measures online. Full psychometric analyses including validity and reliability were conducted.

**Results:**

A final tool was established consisting of three domains related to the burden of leakage: “Emotional impact,” “Usual and social activities,” and “Coping and control.” Convergent validity was evaluated by benchmarking to existing health-related quality of life instruments (domains of SF-36 and Ostomy-Q). This showed high correlation between domains of the leakage tool and other measures, in particular for the Emotional impact domain when compared with SF-36 Emotional well-being and Ostomy-Q Confidence domain (*p* < 0.001). Coping and control correlated moderately well with most PROs tested for except the physical functioning domains, which showed only modest correlation (*p* < 0.001). Usual and social activities correlated equally well with all domains. Internal consistency was high for Emotional impact and Usual and social activities (> 0.92).

**Conclusion:**

The study highlights how users define leakage and its impact in a way that is meaningful to them. This information has been used to develop an instrument to measure leakage which can potentially be used by clinicians and researchers. The instrument demonstrated evidence supporting its reliability and validity as an outcome measure to assess the impact of leakage in stoma care.

**Electronic supplementary material:**

The online version of this article (10.1186/s12955-018-1054-0) contains supplementary material, which is available to authorized users.

## Background

In Europe, it is estimated that some 700,000 people are living with a stoma whilst in the US, approximately 750,000 people have a stoma [1, 2]. A stoma operation can lead to significant changes in a patient’s life and cause difficulties in coping with life with a stoma [3]. The Ostomy Life Study was conducted with over 4000 people with a stoma from 11 different countries, and showed that most of the participants experienced leakage (defined as seeping of output from the stoma under the adhesive of the appliance and/or onto their clothes or bedding) [4].

Leakage can affect a person’s physical and emotional well-being. Studies have shown that frequency of leakage is significantly correlated with the severity of peristomal skin complications [5] and that many people with a stoma do not recognize that they have a skin disorder [6, 7]. The emotional burden of leakage and its impact has also been reported as significant whereby the sudden experience of leakage can be a significant concern for people with a stoma. The Ostomy Life Study showed that 91% of the participants in the survey worried to some degree about leakage and 69% reported that their main reason for worrying was the consequences of leakage. Emotional worry had a great impact on their daily lifestyle and their level of activities [4].

Although the term “leakage” is widely used, there is no clear definition of how to measure the consequences and impact of leakage. Most studies refer to the frequency and severity of self-reported leakage but there is no validated or standardized tool for assessing the subjective and emotional burden of leakage. Currently, two scales are being used to measure leakage: the Dialogue Study Ostomy Appliance Questionnaire [5] and the City of Hope – Quality of Life – Ostomy Questionnaire [8], as well as Likert scales. These measures are not psychometrically validated and do not provide a clear definition of leakage.

The aim of the current study was to get a deeper understanding and definition of leakage through clinical expert input as well as evaluation of people’s experiences of leakage using qualitative methods. The new tool was expected to partly record the actual experience of leakage and then measure its impact on people’s quality of life. The qualitative data were then used to develop and validate a new measurement tool to understand the impact of leakage for people using a stoma appliance, in the UK, US, France and Denmark.

## Methods

### Study design

**Fig. 1:**
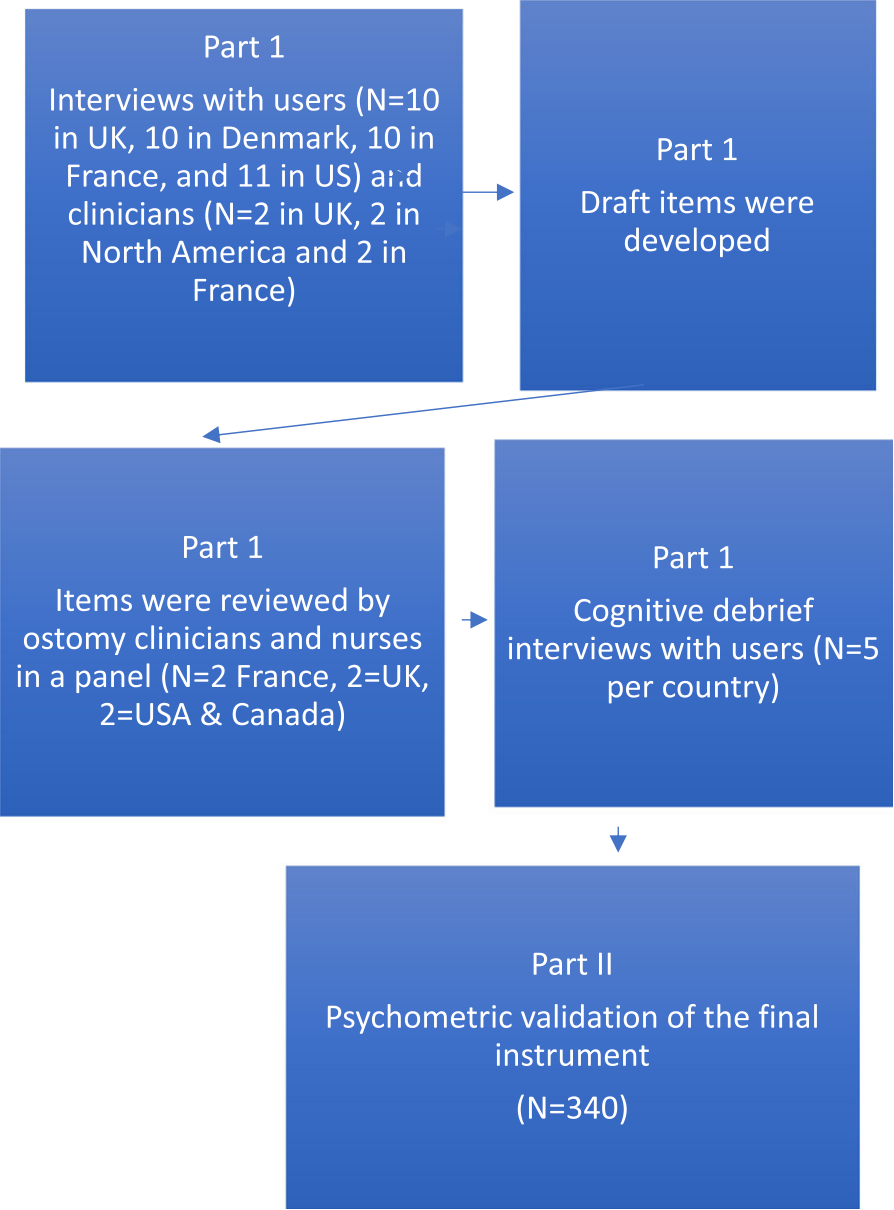
Study design

The study was conducted in two parts. Part 1 involved reviewing the literature to define leakage and develop a new definition with the help of clinical experts, and was conducted in the UK, US, Denmark and France. Part 2 involved the development and psychometric validation of the leakage measure, and was conducted in the UK, US and France (Fig. 1). All participants were eligible if they had a stoma for a minimum of 3 months, were using an ostomy device currently were aged between 18 to 85 years, had access to the internet (Part 2 only), and currently resident in the UK, US, France or Denmark.

Prior to the commencement of main data collection, ethical approval for the study was obtained by the Salus Institutional Review Board in the US (REF: NCL0101). Amendments and translations were sought each time the new instrument was revised. The items were translated using a two-forward/two-backward translation process. Before any data were collected, participants provided consent to participate. All participants were reimbursed for their time by points that they could use in the Coloplast user panel to purchase items.

### Part 1

#### Literature review

A systematic literature review was conducted to understand how leakage is currently defined and experienced by patients and clinical experts and to guide the development of interview guides to explore leakage and its measurement in qualitative interviews. The review was conducted in PubMed and Google Scholar. Search terms included “Ostomy,” “Ostomy/adverse effects,” “Stoma,” “Colostomy,” “Ileostomy,” “Quality of life,” “Patient preference,” and “Patient satisfaction”. Free text search terms were used where MeSH terms were unavailable. These included “Peristomal skin” and “Ostomy leakage.” Findings are reported in the Results section.

#### Qualitative interviews

##### Sample

For the qualitative research, participants were recruited via a Coloplast user panel in each country (*N* = 10 in UK, 10 in Denmark, 10 in France, and 11 in US). Coloplast user panel includes participants who have used or are currently using Coloplast products. They are able to access information, and support through the panel. The sample size was determined by saturation in each country i.e. when interviews were not producing any new information [9]. Eligible participants were contacted via email and provided an outline of the study and an informed consent form. Interviews were conducted by telephone whereby participants were asked about their own experience of leakage, their understanding of leakage, and how it impacts their daily life. The interviews explored all definitions of leakage found in the literature review and also elicited participants’ views regarding what leakage was. Clinicians who specialize in ostomy care were invited to take part in telephone interviews. Two clinicians from the UK, North America, and France (*N* = 6 in total) took part in hour-long interviews that explored how leakage is currently defined, plus its impact on patients’ health-related quality of life (HRQoL), how leakage is and should be measured, and how an improvement in leakage should be defined.

Qualitative interviews were analyzed by two researchers. The data were divided into themes and sub-themes initially by the interview guide and then reviewed against the data. Two researchers reviewed all data and analyzed data according to the themes and sub-themes. Additional themes were added if needed. These were reviewed by a third researcher for final agreement. The findings from the qualitative work were used to draft items that explored all the aspects of HRQoL mentioned by participants. In order to develop items and domains accurately and identify the most relevant areas of impact, a panel of clinical experts was organized. The items were initially developed in English with the aim to translate into relevant languages. The panel aimed to develop items that would be most important for users and also be culturally valid, which would ensure that items were in culturally appropriate language for each country. This panel consisted of six experts, and included ostomy clinicians and nurses (*N* = 2 France, 2 = UK, 2 = North America).

The findings of the qualitative work were presented to the panel. The panel discussed the aspects of leakage that are most important to patients and clinical experts, including the clinicians’ recommendations for a new instrument. Draft items were presented to the panel, including recall period and response options for their feedback. To address limitations of existing instruments, the draft measure included understanding participant’s experience of leakage from predefined options (leakage under the baseplate, leakage onto clothes, leakage due to pouch failure, and other) and frequency of leakage in the last 7 days, as agreed by the panel and the study team. A baseplate adheres to the peristomal (around the stoma) skin, helping protect the skin from stoma output, and attaching the pouch to the body.

Based on the qualitative work, items were developed for four domains (emotional, physical activity, social well-being, and a more general domain which included lifestyle and diet). The study team included these areas as it would capture the most wide areas of functioning. Each domain included positively and negatively worded items and the panel was asked to review and modify items if needed. The draft instrument was reviewed by the team and finalized for testing through two rounds of review.

#### Cognitive debriefing interviews

The testing of the instrument was conducted by doing cognitive debrief interviews with participants in each country. This involved the evaluation of the content validity and patient understanding of the new instrument, before conducting psychometric validation (Part 2). Content validity of the new instrument was evaluated during five cognitive debriefing/exploratory interviews conducted in each country (Denmark, UK, US, and France) with individuals with ileostomy or colostomy using stoma appliances. Participants were sent the instrument beforehand and asked to complete at home prior to the interview. Cognitive debriefing interviews were conducted by telephone in each country to evaluate item comprehension and interpretation, completeness of item coverage, item relevance, and clarity and readability of the new instrument [10]. Among the qualitative methods, cognitive interviewing allows direct input from respondents on the item content, format, and understandability. This method has emerged as an essential component in the development of a number of standardized measures. Participants had the instrument in front of them during the interview. Participants were asked to read each item separately, describe what it means to them, and how they answered it. They were asked if the item was clear, or if it should be revised in any way. Participants suggested removing “negative” statements, such as “I was not able to do things I wanted to,” as these were potentially confusing. Some items were reworded and final revisions were made based on feedback. Some negative items remained as the study team felt there should be a balance of positive and negative items. The items that participants had found confusing were reworded for clarity.

### Part 2: Psychometric validation

Participants who had a stoma and used a stoma appliance were recruited in three countries (up to 200 participants in the UK, and 100 each in France and the US) via the Coloplast CORE panel. This is an online forum maintained by Coloplast to facilitate contact with end-users. Potential participants were sent email invites with a URL link to the study survey, and on entering the survey they completed a study screener to assess their eligibility.

Participants were asked whether they would like to repeat the survey after a 10–20-day period for assessing test-retest reliability. After indicating consent using an IRB-approved consent form, participants completed a sociodemographic/clinical background form and the new leakage instrument. Participants were invited to take part in the retest after 10–20 days.

#### Measures

All participants completed the following sections in the online survey: a sociodemographic and clinical background form, Rand SF-36 (Physical functioning, Role limitations, Social functioning, and Mental health domains [11]), Ostomy-Q instrument (Confidence domain [12]), Ostomy Adjustment Inventory-23 (Anxious preoccupation and Social engagement [13]), and Global ratings for each domain on the instrument. SF-36 is a validated measure that has been used in several patient populations [11]. It consists of eight scaled scores, of which participants completed two four domains. The scale is from 0 to 100, where a lower score indicates worse functioning. The Ostomy-Q is a measure designed to assess quality-of-life outcomes for people using an ostomy. It was validated with ostomy patients [12] and includes four domains, of which participants completed the Confidence domain. The OAI-23 is a validated measure that has been used to measure psychological adjustment in patients with an ostomy. It includes four factors of which two were completed by participants. At baseline and follow-up, participants were also asked four additional global rating type questions regarding the overall impact on their lives of leakage or worry about leakage. The other three questions assessed the specific emotional impact, impact on usual activities, and impact on social activities. These were rated on a 5-point scale (‘not at all’ to ‘very much’), at baseline and follow-up. Those participants who provided the same rating at each time point were considered stable and were included in the assessment of test-retest reliability.

#### Analysis

All instruments were scored and summarized for each participant. Sociodemographic/clinical data were summarized by descriptive analyses as appropriate.

#### Item reduction

Decisions regarding item reduction were based on information from item performance, the principal components analysis (PCA) and the reliability analysis. The plan was to develop a larger set of items than needed, and then through examining item performance the pool of items was reduced by removing those that functioned poorly.

Item variability determines the extent to which the range of potential responses for each item is selected by the participants. The total number of responses, percentage of total responses for each item, and floor and ceiling effects were calculated. The presence of a floor or ceiling effect was defined to occur when 50% of the responses were in the lowest or highest response category for any item [14, 15]. PCA was used to explore the “hypothesized factor structure/domains” of the leakage tool (see Additional file 1). This analysis tested several different issues. The instrument was designed such that each of the four domains had a number of different items reflecting aspects of that domain. The PCA tested the extent to which items loaded into their domains. It also tested whether items loaded into more than one domain (usually considered undesirable). Different approaches to the PCA were used in an attempt to better describe the patterns of variability in the data.

#### Internal consistency

Internal consistency of each conceptual domain was measured using Cronbach’s alpha. A minimum alpha value of 0.70 was considered acceptable [16]. An alpha value between 0.80 and 0.95 indicated that the domain was internally consistent [17].

#### Test-retest reliability

Test-retest reliability was assessed by correlating domain scores between baseline and 10–20 days after baseline, in participants reporting no change in leakage in the global ratings questions. (At reviewer request, we also assessed the test-retest reliability of the global ratings questions for all participants).

**Fig. 2:**
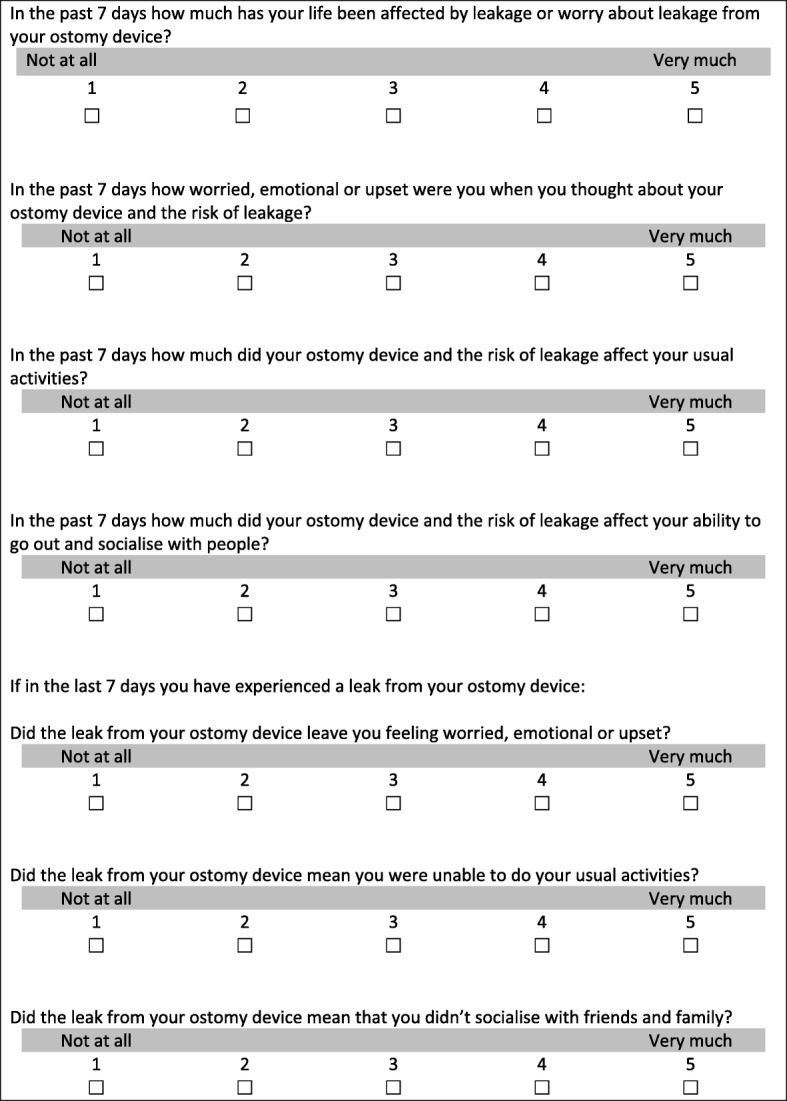
Global ratings to measure change in test-retest reliability

The intraclass correlation coefficient (ICC; two-way mixed, single measure (3,1)) was used to test for test-retest reliability [18]. For the purposes of this study, the following ICC thresholds were used: scale-level ICC > 0.70 was considered acceptable and an ICC > 0.80 was considered good. Questions were also used to identify participants who reported a change in their perception of the overall impact on them of leakage. Participants who reported a change on the global ratings from baseline (either better or worse) were separated out as different groups (Fig. 2; see also Additional file 2). The degree of change in their scores on the leakage tool was calculated and effect sizes and standardized response means were calculated.

#### Convergent and concurrent validity

Convergent validity of the new instrument was evaluated by benchmarking the new instrument with two validated instruments: the total score of the OAI-23 and domains of SF-36 (Emotional functioning, Physical functioning, Role physical, and Social functioning), and Ostomy-Q (Confidence domain).

Associations between these measures were explored using parametric and/or non-parametric correlations as appropriate. Convergent validity was considered supported if correlation coefficients between related scales were > 0.40. More specifically, for the Emotional impact domain we hypothesized correlations with total score (OAI-23); and Emotional well-being (but not Physical functioning or Role physical) (SF-36) and the Confidence domain (Ostomy-Q). The Usual and social activities domain was hypothesized to correlate with total score (OAI-23) and Physical functioning, Role physical, and Social functioning domains (SF-36). Coping and control was hypothesized to correlate with total score (OAI-23) and Confidence domain (Ostomy-Q), but not with Physical functioning (SF-36).

#### Minimal important differences

Different methods exist for determining the degree of change that would be considered a minimal important difference. These values support the interpretation of change data. The methods used here are based on statistical dispersion (standard error of measurement and ½ standard deviation) and degree of change indicated by the global ratings.

## Results

### Part 1

#### Literature review

Of 213 titles, 11 full articles were considered relevant. The overall review of the peer-reviewed literature identified no standard accepted definition of “leakage.” In some studies, participants were asked to report experience of leakage without definition [5]. When described, the literature stated two general areas of leakage measurement: leakage “under” the baseplate and/or leakage “outside” the baseplate [5, 7, 19].

Two scales to measure leakage were identified: the Dialogue Study Ostomy Appliance Questionnaire [5] and the City of Hope – Quality of Life – Ostomy Questionnaire [8]. Other studies reported using subjective Likert scales to measure frequency or severity of leakage. Although these measures were used, there was no clear validation of the questions applied to measure leakage in a systematic way.

#### Qualitative interviews

In the qualitative interviews, users described leakage in several ways: “when it has reached my clothes,” “stool leaking out from under the plate,” “leakage under the barrier or baseplate,” or “when it goes onto my skin (from under the skin barrier).” Participants were asked to describe the impact of leakage in four areas: emotional well-being, social, physical activity, and lifestyle. Participants described a “fear of leakage,” which felt as if they had loss of control. Even if participants hadn’t actually experienced leakage, the possibility of it would always be present. Due to the fear of leaks or experience of actual leaks, people felt embarrassed in public places, and shame and worry that leakage could happen at any time. Participants expressed feeling isolated and withdrawn if leakage occurred. A small number of people felt socially limited: most would go out regardless of fear of leakage. Some participants reported planning ahead for a specific activity such as travel or sport. People felt that they could not do certain activities such as swimming or golf, due to fear of leakage. However most people managed their condition and didn’t let their ostomy limit their activities. Some also expressed limitations in diet and having to wear loose clothing due to leakage.

The clinicians (*N* = 6) also reported similar impacts of leakage. According to clinicians, in their experience most people with stomas defined leakage in terms of when they had to change clothing. However, they also were clear that there was variability in how people defined it. The clinicians reported that many users experienced social isolation, lack of self-esteem, and difficulty in intimacy with their partners due to leakage or the fear of leakage. Clinical experts expressed concern that there are few valid tools to measure the impact of leakage. They suggested that a multifaceted measure should be developed to measure objective leakage, through use of photographs, and also the subjective impact of leakage. In addition, clinicians believed that the impact of leaks must be measured in the following areas: psychological impact, coping skills, physical activity, and confidence.

#### Sample

In total, 340 people took part in the survey, but most analyses are based on a smaller sample as not all participants completed all questions. Participants’ demographic and background characteristics are given in Table 1.

**Table 1:** Sociodemographic profile of sample at baseline

	Country			
	UK	US	France	Total
Sex				
Male (*n*,%)	75 (52.4)	42 (32.3)	38 (56.7)	155 (45.6)
Female (*n*,%)	68 (47.6)	88 (67.7)	29 (43.3)	185 (54.4)
Age (mean, SD)	57.5 (14.0)	60.5 (12.5)	61.2 (12.6)	59.4 (13.2)
Colostomy				
Yes (*n*, %)	55 (38.5)	49 (37.7)	43 (64.2)	147 (43.2)
Ileostomy (*n*, %)	89 (62.2)	82 (63.1)	25 (37.3)	196 (57.6)
In the last 7 days, have you experienced leakage? Yes (*n*,%)	73 (51.0)	58 (44.6)	28 (41.8)	159 (46.8)
Leakage under the baseplate only (*n*,%)	48 (33.6)	36 (27.7)	22 (32.8)	106 (31.2)
Leakage outside the baseplate (*n*,%)	43 (30.1)	33 (25.4)	17 (25.4)	93 (27.4)

#### Testing the conceptual framework and item reduction

The PCA showed that the proposed conceptual framework for the measure was not well supported. It was therefore agreed that the PCA should guide the development of a new conceptual framework that could be supported in terms of item content and statistical analysis. In the PCA analysis, factor solutions were forced or were allowed to emerge. Different approaches to rotation were explored, and after several rounds the use of promax rotation was settled on. Various methods of factor analysis were also explored, such as principal axis factoring. Items were removed if they cross-loaded (loaded above 0.400 on two different domains). Items were also removed if they did not appear to fit a domain conceptually. Through this process, 14 items were removed from the measure, leaving 22 items. The original four domains were not supported by the PCA. A three-factor solution was considered to be the best fit for describing the data. The pattern of missing data meant that the final PCA only included data from 93 participants, and so this analysis was repeated with a mean imputed value (as opposed to list-wise deletion) [20]. The final PCA results were very similar in both models.

**Fig. 3:**
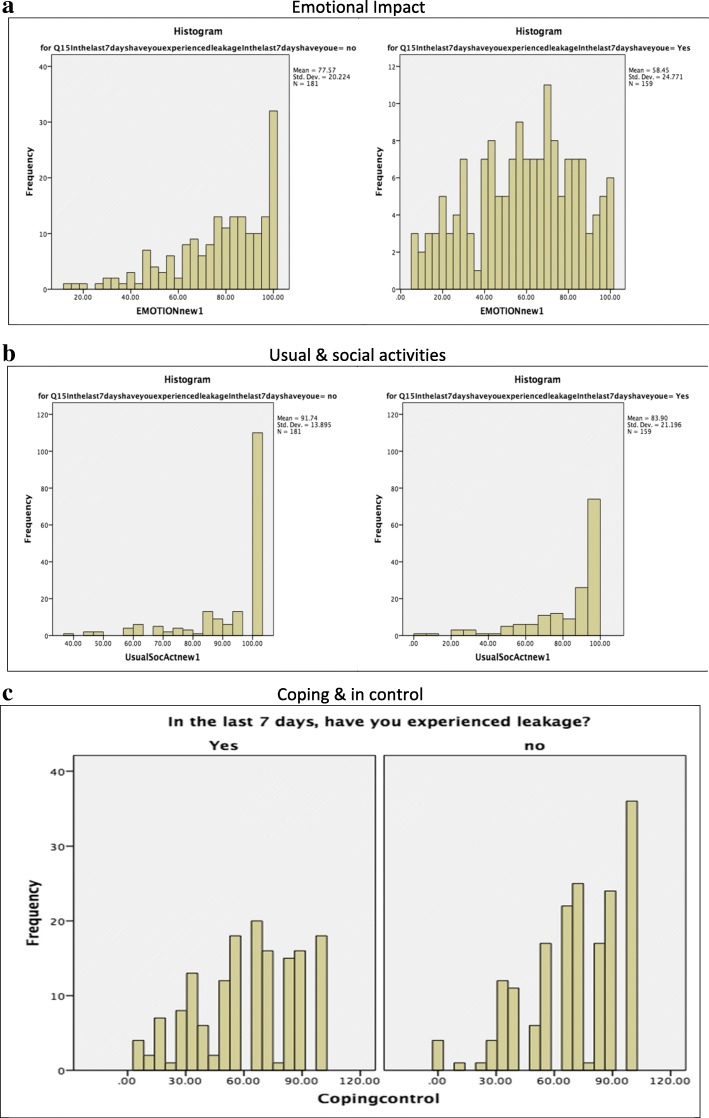
Distribution of scores indicating whether a leak was experienced in the 7 days before baseline. The leakage tool included the proposed domains **a** Emotional impact, **b** Usual and social activities, and **c** Coping and control

**Fig. 4:**
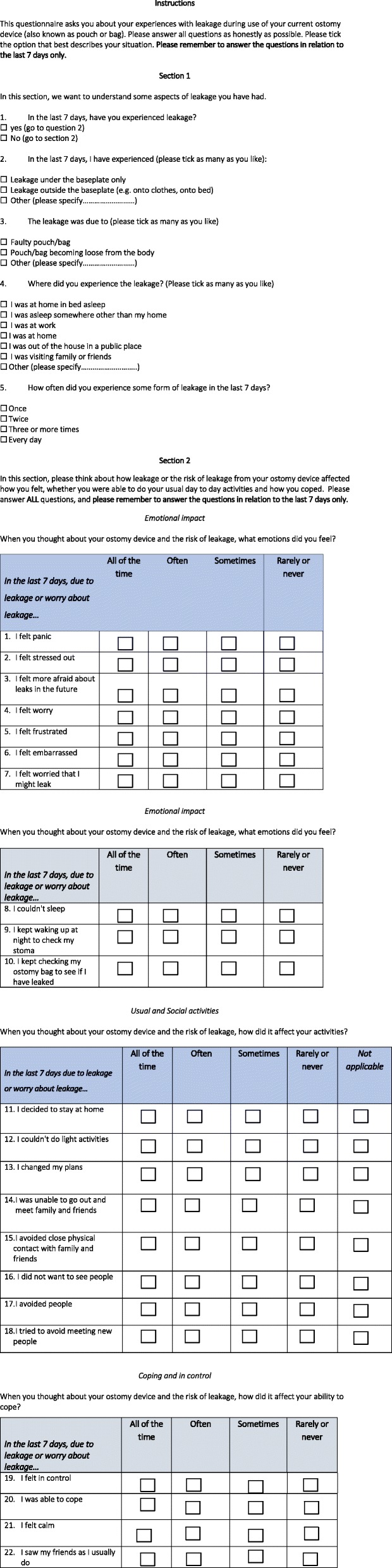
The final measure-to-measure burden of leakage (Ostomy Leak Impact Tool)

These three factors were labeled Emotional impact; Usual and social activities; Coping and control. Some of the items had quite a high proportion of responses at the extremes, but these items were retained based on other aspects of their performance (data not shown). Many items in the Usual and social activities domains were removed, and what was left seemed to suggest a grouping of items around a single concept that reflected everyday or usual activities and included social activities. Lastly, a different set of items emerged that appeared to reflect issues related to coping and feelings of being in control. This led to some analyses to determine whether these domains are measuring something of importance or concern. To explore this, the distribution of the data for people who reported that they had experienced a leak in the last 7 days was compared with those who hadn’t (Fig. 3a for a histogram of this analysis for the Emotional impact domain). More detailed histograms are presented in Additional file 3. The box plots for the Emotional impact domain indicate that the scores are reasonably normally distributed. The box plots of the Usual and social activities domain (Fig. 3b) suggest a degree of skew in the data. There is a difference between the two groups in terms of distribution, but the scores in both groups are very high. Figure 3c shows the box plots for the Coping and control domain, which shows evidence of normally distributed scores and sensitivity to the experience of a recent leak. The histograms in Additional file 3 unpack these data in more detail. These analyses suggest that there are some limitations in the distribution of the Usual and social activities domain, but the other two domains seem to show normal patterns of distribution. The final tool is shown in Fig. 4.

#### Internal consistency

The internal consistency estimates ranged from 0.77 (Coping and control) to 0.93 (Emotional impact). The internal consistency of the Coping and control domain may be slightly lower because the domain includes fewer items (Table 2).

**Table 2:** Test-retest reliability, based on those people who report no change between baseline and follow-up on global ratings

	ICC	95% CI	*R*
Global rating Q1 – no change between baseline and follow-up (*N* = 169)			
Emotional impact	0.885	0.847–0.913	0.885
Usual and social activities	0.814	0.756–0.859	0.814
Coping and control	0.582	0.472–0.673	0.583

#### Test-retest reliability

In the global ratings, most participants reported the same answer at both time points, and the distribution of people who changed responses was fairly even (data not shown). The data suggest that the leakage measure is fairly stable over time (Table 2) and that the majority of participants didn’t report any change between baseline and follow-up (*N* = 169). The test-retest reliability (ICC) of the global rating questions all exceeded 0.70.

#### Criterion validity

The validity of the three subscales of the leakage questionnaire was examined against different external criteria. These criteria included the experience of leakage, how frequently people had leaked in the previous 7 days, where the leak had happened, and the nature of the leak itself – under the baseplate only, or onto clothes. Table 3 shows the impact of a leak in the last 7 days on participants’ scores on the three dimensions of leakage tool. All three were significantly different.

**Table 3:** Test for differences in scores on the three domains of the leakage tool

		*N*	Mean	*t*	*p-*value
In the last 7 days, have you experienced leakage?					
Emotional impact	Yes	159	58.4	−7.831	< 0.001
	No	181	77.6		
Usual and social activities	Yes	159	83.9	−4.077	< 0.001
	No	181	91.7		
Coping and control	Yes	159	62.4	−3.234	0.001
	No	181	71.3		

Contrasting patients who have or have not experienced leakage in the last 7 days

Further analysis was conducted that explored the impact of leaks by location, i.e. whether the location of the participant at the time of the leak has a significant effect on a participant’s subjective experience. The data show that whether participants experienced a leak at home or not has very little impact on the scores on the leakage tool (Table 4). In contrast, those who report having had a leak in a public place outside the home report significantly lower scores on the Coping and control domain and a difference on the Usual and social activities domain that approaches significance (*p* < 0.05). For the Emotional impact domain, the trend was the same. In addition, the impact of the severity of most recent leak experienced by the participant was also explored. This was classified as either none, leakage under the baseplate only, leakage onto clothes, or leakage under the baseplate and onto clothes. All three domains were sensitive to differences between the patients, grouped in terms of leak severity. The most marked effect seemed to be related to the occurrence of leakage onto clothing (Table 5).

**Table 4:** Impact of where the leak happened, either at home or in a public place

		*N*	Mean	*t* (*p*-value)
I was at home				
Emotional impact	Yes	70	58.4	NS
	No	89	58.5	
Usual and social activities	Yes	70	83.2	NS
	No	89	84.5	
Coping and control	Yes	70	63.1	NS
	No	89	61.8	
I was outside the home in a public place				
Emotional impact	Yes	43	53.5	−1.54 (NS)
	No	116	60.3	
Usual and social activities	Yes	43	78.6	−1.93 (0.056)
	No	116	85.9	
Coping and control	Yes	43	54.6	−2.30 (0.02)
	No	116	65.3	

*NS* Not significant

**Table 5:** Impact of leak severity on scores on the three domains of the leakage tool

	Nature of leak	*N*	Mean	SD	95% CI	*F*	*p-*value
Emotional impact	None	186	77.0	20.6	74.0–80.0	22.4	< 0.001
	Leak under baseplate only	61	65.5	23.4	59.5–71.5		
	Leak onto clothes	48	54.8	25.9	47.3–62.3		
	Under b/plate and onto clothes	45	53.1	23.7	46.0–60.2		
Usual and social activities	None	186	91.5	14.2	89.5–93.6	8.32	< 0.001
	Leak under baseplate only	61	89.2	13.7	85.6–92.7		
	Leak onto clothes	48	79.8	25.2	72.5–87.1		
	Under b/plate and onto clothes	45	81.2	23.9	74.0–88.4		
Coping and control	None	186	71.0	24.8	67.5–74.6	5.98	< 0.001
	Leak under baseplate only	61	69.6	23.2	63.7–75.6		
	Leak onto clothes	48	57.6	30.1	48.9–66.3		
	Under b/plate and onto clothes	45	57.8	24.8	50.4–65.3		

*SD* Standard deviation

#### Concurrent validity

The performance of the three domains of the leakage tool was compared and benchmarked against other established measures of general HRQoL- and ostomy-related confidence, adjustment, and coping. The hypothesized relationships between measures (Table 6) was well supported. All hypothesized relationships exceeded the criterion of a correlation > 0.40; however, a number of relationships that were not predicted were also found – such as correlations between Emotional impact, Social functioning, and Coping and control. Emotional impact also correlated with Physical function (SF-36), which didn’t match our hypotheses.

**Table 6:** Correlation indices (Pearson’s r) between the leakage tool and other measures (SF-36, Ostomy-Q and OAI-23)

	SF-36 Physical functioning	SF-36 Role physical	SF-36 Emotional well-being	SF-36 Social functioning	Ostomy-Q Confidence	Ostomates Adj Inv-23 Total
Emotional impact	*0.424*	0.461	**0.605**	0.525	**0.771**	**0.730**
Usual and social activities	**0.536**	**0.472**	0.575	**0.602**	**0.587**	**0.660**
Coping and control	*0.253*	0.299	0.486	0.477	**0.442**	**0.480**

All correlations at *p* < 0.001

Bold numbers are predicted relationships, italicized numbers were predicted to be unrelated

#### Sensitivity

Numerical estimates of sensitivity were estimated (Table 7) based upon the degree of change from baseline to follow-up in the leakage tool for those people who reported some level of improvement on the global ratings. The variables that were estimated include effect size. Effect size estimates suggested that the Emotional impact domain was the most sensitive. In contrast, Coping and control showed little sensitivity to self-reported change.

**Table 7:** Numerical estimates of sensitivity based on dispersion – effect size and SRM and estimates of MID

Domain	Improvement		Decline		SEM	½ SD	Global rating anchor
	Effect size	SRM	Effect size	SRM			
Emotional impact	0.39	0.52	−0.26	−0.49	5.4	10.4	7.0
Usual and social activities	0.26	0.40	−0.10	−0.12	5.9	10.3	3.5
Coping and in control	−0.01	−0.01	−0.13	− 0.13	9.9	10.9	0.65

Based on all methods

*MID* Minimal important difference, *SD* Standard deviation, *SRM* Standardized response mean

#### Minimal important difference

The degree of change that participants considered an important difference on the scale was estimated using a range of standard methods (as outlined above). The scores on the leakage tool reflected the improvement or deteriorations of users. There was an interesting effect whereby those reporting no change were also those reporting the least burden from leaks (data not shown). Table 7 shows the minimal important differences based on three methods.

## Discussion

This study evaluated how people define leakage in ostomy care through the use of qualitative research in order to develop and validate a patient-reported leakage tool for use in clinical research. A valid and reliable scale allows clinicians and users to understand the efficacy of an ostomy device in terms of improvement in leakage. This is the first study to explore and define the impact of leakage in the US, UK, France and Denmark. The study was conducted in two parts: 1) qualitative work, to understand how users define leakage and develop an instrument; and 2) psychometric validation of the instrument in the US, UK, and France. The psychometric validation was conducted for the English and French versions of the instrument. The current instrument was designed to provide a validated and reliable measure for this area where there is currently no tool specifically designed to assess the subjective impact of leakage. The work resulted in a tool which captures the actual experience of leakage (factual section) and then assesses the impact of leakage in three domains (Emotional-Impact’, ‘Usual-and-Social activities’, and ‘Coping-and-Control’). The instrument is translated into three languages for US, UK and France.

The qualitative work showed the variation in how people define leakage related to their ostomy device. The literature and users regard leakage as “output from the stoma” onto the baseplate, or leakage that soils clothes or bedding [5]. Each type of leakage may have a different impact on HRQoL, and therefore the lack of an established definition could limit our ability to measure the impact of leakage in a systematic and valid way in clinical research. The current work has provided suggestions for establishing a definition of leakage in the future – such as “leakage under the baseplate” or “leakage outside the baseplate (onto clothing, onto bed),” with clear definitions of what leakage could mean for the user. In addition, by asking users to describe which leakage they experience, the current tool address the limitations of previous measures which have unclear definitions. The previous measures do not provide a specific definition, and therefore there is some ambiguity about the user’s perception of leakage.

The qualitative work with users and clinicians revealed that leaks can impact people in varied ways and that not only was there an impact of an actual leak but people also feared potential leaks. The fear of leakage led to feelings of embarrassment and loss of control. Participants expressed strong emotions such as anger, panic, and frustration due to the need to constantly check for leaks; and a leak could lead to constant worry and social isolation. The evidence shows that there is great impact of leakage on emotional well-being, which previous literature has not reported in such detail.

The measure was also able to show the impact of leakage by frequency of leakage, where the leak had happened, and the nature of the leak itself (under the baseplate only, or onto clothes). As frequency of leakage increased, there was a greater impact on each domain score, i.e. people worried more with increasing leakage, which is also supported by the findings of Erwin-Toth et al. [21]. Participants also indicated that a leak at home had less impact than a leak in a public place. This is supported by the earlier qualitative work in which users reported feeling panic and fear in public places if they experienced a leak. In addition, participants reported the most impact in these domains when leakage occurred onto clothing – which is not surprising.

Minimal important difference estimates were also calculated in order to assess a meaningful change for users. These estimates may be useful in measuring a meaningful change for people who use ostomy devices in clinical trials.

The psychometric validation has shown that this instrument could be used to assess the nature of leaks and the impact of leakage on HRQoL among users of ostomy devices. Previous measures mainly evaluated leakage in terms of frequency and severity, whereas the current tool assesses the user’s experience of leakage and consequently its impact on emotional well-being and usual activities and coping. This is an important addition to the field as it is also supported by clinician feedback, and hopefully it will prove to be a useful tool in measuring the efficacy of devices or for benchmarking the well-being of people living with a stoma. The measure was able to show responsiveness in various domains at different time points, perhaps suggesting that leakage has varying impact on domains.

This study had some limitations, which should be considered. The sample was recruited from the Coloplast CORE panel, which indicated that most patients had used Coloplast products at some stage. This may mean that the study sample is not completely representative of people who use ostomy devices. A different grouping of items emerged from the analysis which on review suggested a slightly different set of domains. This may not be a limitation as such, but it does suggest that further exploration of items could be done. The estimation of test-retest reliability was itself reliant upon people’s responses to a global rating designed to test whether they believe that they had changed in this regard (e.g. Coping and control) since the baseline assessment. Only those participants who reported no change were included in the analysis of test-retest reliability. However, this analysis is dependent on these unvalidated and untested global ratings, which is a significant limitation. This criticism should also be considered when examining the data regarding sensitivity to change and the minimal important difference. These data are also dependent on the robustness of the global ratings. Further research should include validation in other countries and should aim to show the strength of these domains in evaluation of impact of leakage in clinical trials.

## Conclusion

The study highlights leakage related aspects important for the well-being of people living with a stoma. Furthermore, the study introduces possible ways to define leakage that can be used in the clinical community and study setup. The leakage instrument demonstrated evidence supporting its reliability and validity as an outcome measure to assess the impact of leakage in stoma care. Further usage and evaluation of the tool in clinical trials and market surveys will be necessary to prove its responsiveness for interventional changes.

## Additional files

Additional file 1: Final PCA model that was used to understand the relationship between items and domains in the survey (loadings less than 0.20 are not shown). (DOCX 53 kb)

Additional file 2: Global ratings to measure change in test-retest reliability. (DOCX 20 kb)

Additional file 3: Distribution of scores on the proposed three domains of the leakage tool: (a) Emotional impact, (b) Usual and social activities, (c) Coping in control. (DOCX 53 kb)

## Abbreviations

HRQoL: Health-related quality of life
ICC: Intraclass correlation coefficient
PCA: Principal components analysis
SF-36: Short-Form survey 36-item
