# Supplementary material for: The ostomy leak impact tool: development and validation of a new patient-reported tool to measure the burden of leakage in ostomy device users
Source: Health Qual Life Outcomes. 2018 Dec 14;16:231. doi: 10.1186/s12955-018-1054-0 (PMC6295083; doi:10.1186/s12955-018-1054-0)
Supplement: Supplementary file 2 — Global ratings to measure change in test-retest reliability. (DOCX 20 kb) [file 12955_2018_1054_MOESM2_ESM.docx]

**Additional file 2: Global ratings to measure change in test-retest reliability**

| In the past 7 days how much has your life been affected by leakage or worry about leakage from your ostomy device?   \| Not at all \|  \|  \|  \| Very much \| \| --- \| --- \| --- \| --- \| --- \| \| 1 \| 2 \| 3 \| 4 \| 5 \| \| ☐ \| ☐ \| ☐ \| ☐ \| ☐ \|   In the past 7 days how worried, emotional or upset were you when you thought about your ostomy device and the risk of leakage?   \| Not at all \|  \|  \|  \| Very much \| \| --- \| --- \| --- \| --- \| --- \| \| 1 \| 2 \| 3 \| 4 \| 5 \| \| ☐ \| ☐ \| ☐ \| ☐ \| ☐ \|   In the past 7 days how much did your ostomy device and the risk of leakage affect your usual activities?   \| Not at all \|  \|  \|  \| Very much \| \| --- \| --- \| --- \| --- \| --- \| \| 1 \| 2 \| 3 \| 4 \| 5 \| \| ☐ \| ☐ \| ☐ \| ☐ \| ☐ \|   In the past 7 days how much did your ostomy device and the risk of leakage affect your ability to go out and socialise with people?   \| Not at all \|  \|  \|  \| Very much \| \| --- \| --- \| --- \| --- \| --- \| \| 1 \| 2 \| 3 \| 4 \| 5 \| \| ☐ \| ☐ \| ☐ \| ☐ \| ☐ \|   If in the last 7 days you have experienced a leak from your ostomy device:  Did the leak from your ostomy device leave you feeling worried, emotional or upset?   \| Not at all \|  \|  \|  \| Very much \| \| --- \| --- \| --- \| --- \| --- \| \| 1 \| 2 \| 3 \| 4 \| 5 \| \| ☐ \| ☐ \| ☐ \| ☐ \| ☐ \|   Did the leak from your ostomy device mean you were unable to do your usual activities?   \| Not at all \|  \|  \|  \| Very much \| \| --- \| --- \| --- \| --- \| --- \| \| 1 \| 2 \| 3 \| 4 \| 5 \| \| ☐ \| ☐ \| ☐ \| ☐ \| ☐ \|   Did the leak from your ostomy device mean that you didn’t socialise with friends and family?   \| Not at all \|  \|  \|  \| Very much \| \| --- \| --- \| --- \| --- \| --- \| \| 1 \| 2 \| 3 \| 4 \| 5 \| \| ☐ \| ☐ \| ☐ \| ☐ \| ☐ \| |
| --- | --- | --- | --- | --- | --- | --- | --- | --- | --- | --- | --- | --- | --- | --- | --- | --- | --- | --- | --- | --- | --- | --- | --- | --- | --- | --- | --- | --- | --- | --- | --- | --- | --- | --- | --- | --- | --- | --- | --- | --- | --- | --- | --- | --- | --- | --- | --- | --- | --- | --- | --- | --- | --- | --- | --- | --- | --- | --- | --- | --- | --- | --- | --- | --- | --- | --- | --- | --- | --- | --- | --- | --- | --- | --- | --- | --- | --- | --- | --- | --- | --- | --- | --- | --- | --- | --- | --- | --- | --- | --- | --- | --- | --- | --- | --- | --- | --- | --- | --- | --- | --- | --- | --- | --- | --- |
