# Supplementary figures and images for: The ostomy leak impact tool: development and validation of a new patient-reported tool to measure the burden of leakage in ostomy device users
Source: Health Qual Life Outcomes. 2018 Dec 14;16:231. doi: 10.1186/s12955-018-1054-0 (PMC6295083; doi:10.1186/s12955-018-1054-0)

**Additional file 3:**  Distribution of scores on the proposed three domains of the leakage tool.


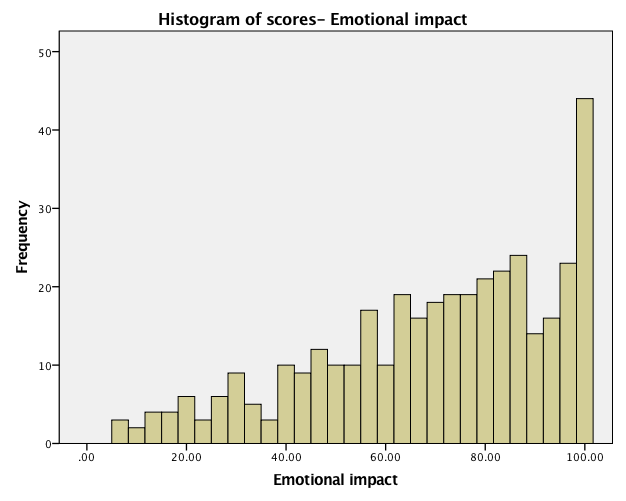


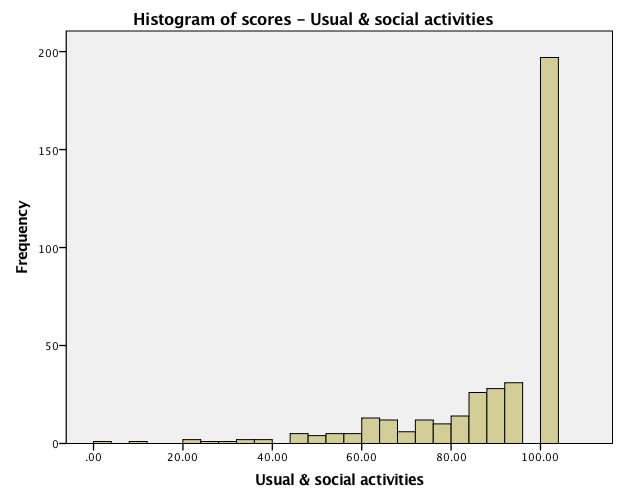


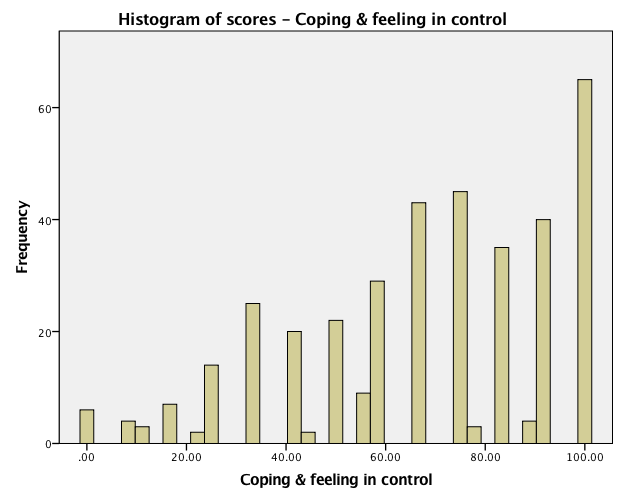

Supplement: Supplementary file 3 — Distribution of scores on the proposed three domains of the leakage tool: (a) Emotional impact, (b) Usual and social activities, (c) Coping in control. (DOCX 53 kb) [file 12955_2018_1054_MOESM3_ESM.docx]
